# Epidemiology of tuberculosis in foreign students in Japan, 2015–2019: a comparison with the notification rates in their countries of origin

**DOI:** 10.1017/S0950268821001977

**Published:** 2021-08-20

**Authors:** M. Ota, T. Nishimura, K. Uchimura, S. Hirao

**Affiliations:** 1 Research Institute of Tuberculosis, Tokyo, Japan; 2 Keio University Health Center, Tokyo, Japan

**Keywords:** Epidemiology, immigrants, Japan, students, tuberculosis

## Abstract

Tuberculosis (TB) in immigrants is becoming a challenge in eliminating TB in Japan. We investigated the epidemiology of TB in foreign students in Japan in 2015–2019. A total of 2007 foreign students with TB whose median age was 22.5 years (1243 (61.9%) were males) were registered. The notification rates peaked in 2016 at 164.0 per 100 000 population and decreased towards 2019. Of the 2007, 535 were from Vietnam, 444 from China and 395 from Nepal. The notification rates were 596.6 per 100 000 person-years (PYs) for Myanmar, 595.4 for the Philippines and 438.6 for Cambodia. The rates were much higher than those of the general populations in their countries of origin for Myanmar, the Philippines, Cambodia, Indonesia, Nepal, Mongolia, Vietnam and China. In comparison with the years 2010–2014, the notification rates for foreign students decreased for the students from Nepal, Vietnam and China. The TB notification rate of the foreign students in Japan can be a good surrogate indicator for the risk of TB among the immigrant subpopulation in Japan and should continuously be monitored. Those who are at higher risk of TB may be annually screened for TB to prevent TB outbreaks.

## Introduction

Japan has successfully reduced the burden of tuberculosis (TB) in the past seven decades from 590 684 cases (698 per 100 000 population) in 1951 to 14 460 cases (11.5 per 100 000 population) in 2019 [1, 2]. However, about 5000 cases of sputum smear-positive pulmonary TB are still reported annually, and these potentially infectious TB cases pose a public health threat to the community [2]. Imported TB from immigrants is also a challenge in eliminating TB in Japan, as seen in Europe and North America [3–5], because Japan has an increasing number of immigrants (2.2 million at the end of 2015), mainly from Asia, namely China, South Korea and Vietnam [6]. The proportion of immigrants among all TB cases in Japan has steadily increased from 2.4% in 2000 to 10.7% in 2019 [2]. Immigrants accounted for 73% of the TB cases among those aged 20–29 years in 2019 [2]. The proportion of multidrug-resistant (MDR) TB, which is resistant to the two most potent anti-TB drugs, rifampicin and isoniazid, in previously untreated all types of TB in Japan was 0.4% in 2019; however, for those born in foreign countries the proportion was much higher (2.8%) [2]. TB outbreaks have involved hospitals, workplaces, military training, schools and sometimes homeless persons [7–13], and an MDR-TB outbreak involving the immigrant population was also reported [14]. Pre-entry screening for TB for immigrants to Japan is expected to be initiated soon [15]. However, the question remains: who, or immigrants from which countries, are to be screened for TB?

The authors previously reported TB in foreign students in Japan from 2010 through 2014 and found the notification rates for those from Myanmar, the Philippines, Nepal and Mongolia were quite high over 200 per 100 000 person-years (PYs) [16]. The risks of TB among immigrants can roughly be estimated using the national TB notification rates of the countries of origin of the immigrants. However, there are several challenges and the estimates may be too low because the national TB programmes (NTPs) of countries may be unable to capture substantial proportions of their TB patients either due to lack of technical or managerial capacities or the private sector simply not reporting patients to the NTPs [17–19]. The risk of TB among immigrants can also be estimated using the numbers of TB patients among the immigrants and the immigrant population in Japan. However, such estimation cannot escape limitations in relation to those who temporarily stay without visas due to the visa waiver programme and the existence of an undocumented immigrant population [20, 21]. To the best of our knowledge, it is only possible to more accurately determine the TB notification rates for foreign students in Japan because the TB surveillance system records occupations, and foreign students (student visa holders) are surveyed by the Japan Student Services Organization (JASSO) annually [22]. This study aims to update the epidemiology of TB in foreign students in Japan using the national TB surveillance and the population statistics data for the foreign students in Japan from 2015 through 2019.

## Methods

The methods of this study were almost the same as in our previous study [16]. Briefly, the study period was the 5 years from 2015 through 2019 and the study population consisted of all the foreign students who stayed in Japan during the study period, including long- and short-term (with a stay of less than 1 year) students. The study included students from 15 countries: Bangladesh, Cambodia, China, India, Indonesia, Malaysia, Mongolia, Myanmar, Nepal, the Philippines, the Republic of Korea, Sri Lanka, Taiwan, Thailand and Vietnam. Other foreign countries were grouped together as ‘others’ as no other country had more than three TB cases in the study period or the number of students from those countries was too small (<300 students) to be listed in the JASSO survey.

The information on the foreign students with TB disease who were registered in the study period was retrieved from the National Epidemiological Surveillance of Infectious Diseases (NESID) system of Japan [23]. The information retrieved from the NESID included the patient's age, sex, year of registration, mode of detection and country of origin. The identification information of TB patients is not normally entered into the NESID. The number of foreign students in Japan in the study period was obtained from JASSO [22]. The TB notification rates were calculated as the number of foreign students with TB disease registered divided by the number of foreign students staying in Japan by country.

The national TB notification data of the countries in the study period were obtained from the World Health Organization [24]. TB notification data for Taiwan were obtained from the Centers for Disease Control, Department of Health of Taiwan [25]. Population estimates of countries were obtained from the United Nations Population Division [26].

Statistical tests, including calculations of 95% confidence intervals (CIs), were conducted using R (Ver x64 4.0.2. The R Foundation for Statistical Computing, Vienna, Austria). Fisher's exact test was employed for comparisons of proportions. Pearson's correlation analysis was used for the associations between two sets of values. *P* < 0.05 was considered statistically significant.

This investigation did not need an ethical review because it used national and global infectious disease surveillance data, and the data concerning the numbers of foreign students in Japan had already been published, and did not involve patients' confidential information.

## Results

There were 2007 foreign students with TB disease registered from 2015 through 2019 with a median age of 22.5 years. Of them, 1243 (61.9%) were males. The characteristics of the foreign students with TB disease are shown in Table 1. Most students (1106, 55.1%) were diagnosed with TB through health screening, though 680 (33.9%) sought health care. From 2015 through 2019, the gross numbers of long- and the short-term foreign students were about 1.33 million and 86 600, respectively.

**Table 1.** Overview of foreign students with tuberculosis disease in Japan, 2015–2019

Characteristics	*n*	%
Age (years)		
18–22	883	44.0
23–27	804	40.1
>27	320	15.9
Male	1243	61.9
Mode of detection		
Health screening	1106	55.1
Self-health-seeking at hospitals/clinics	680	33.9
Other	221	11.0
Total	2007	100

**Fig. 1.**
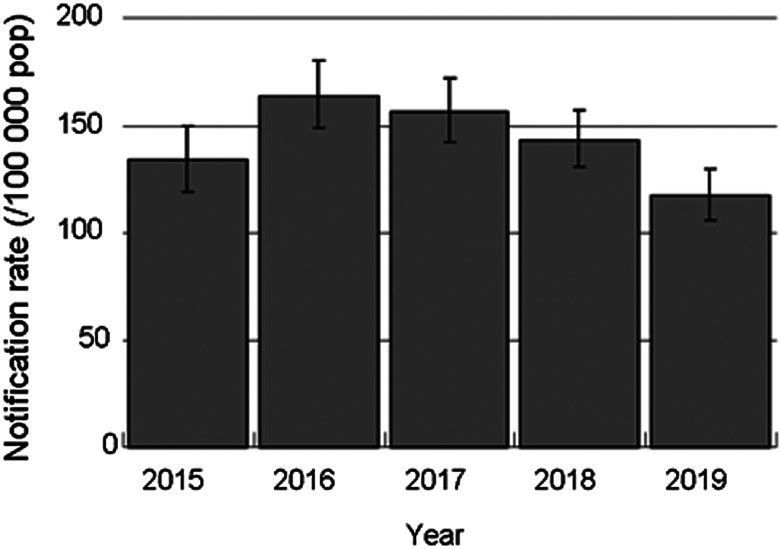
Trend of tuberculosis notification rates in foreign students, Japan, 2015–2019. pop = population.

Figure 1 shows the trend of the TB notification rates among the foreign students. From 2015 to 2016, the notification rate increased from 133.5 (95% CI 118.8–149.6) per 100 000 population to 164.0 (95% CI: 148.7–180.5) per 100 000 population and then decreased to 117.5 (95% CI: 106.1–129.8) per 100 000 population in 2019.

**Fig. 2.**
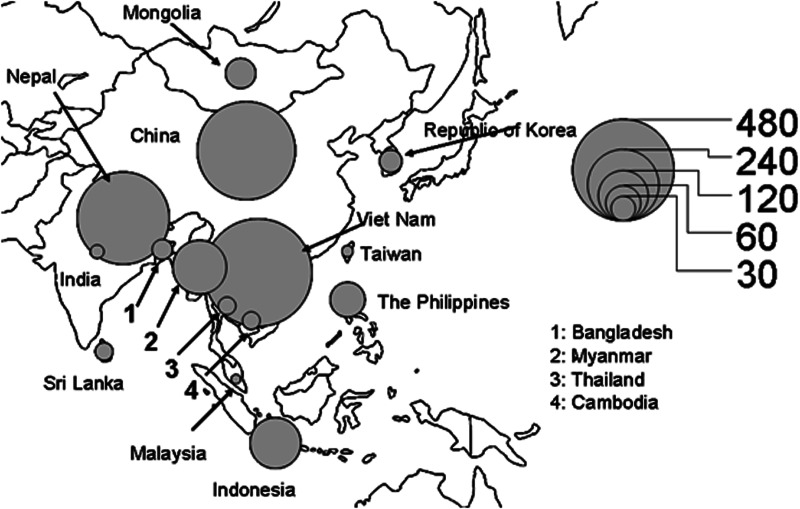
Geographic distribution of countries of origin of foreign students with tuberculosis, Japan, 2015–2019.

Figure 2 gives the geographic distribution of the countries of origin of the foreign students with TB disease from 2015 to 2019. Five hundred thirty-five (26.7%) were from Vietnam, 444 (22.1%) from China, 395 (19.7%) from Nepal, 137 (6.8%) from Myanmar and 122 (6.1%) from Indonesia.

The TB notification rate for male foreign students was 168.0 (95% CI 158.8–177.6) per 100 000 PYs, whereas that for females was 130.9 (95% CI 121.8–140.5) per 100 000 PYs. Since disaggregated numbers of the sexes of the short-term foreign students were not available, the numbers of the short-term foreign students (about 6% of the numbers of the total foreign students [22]) were not included in the calculation for the TB notification for each sex. Therefore the TB notification rates for males and females were a little overestimated.

**Fig. 3.**
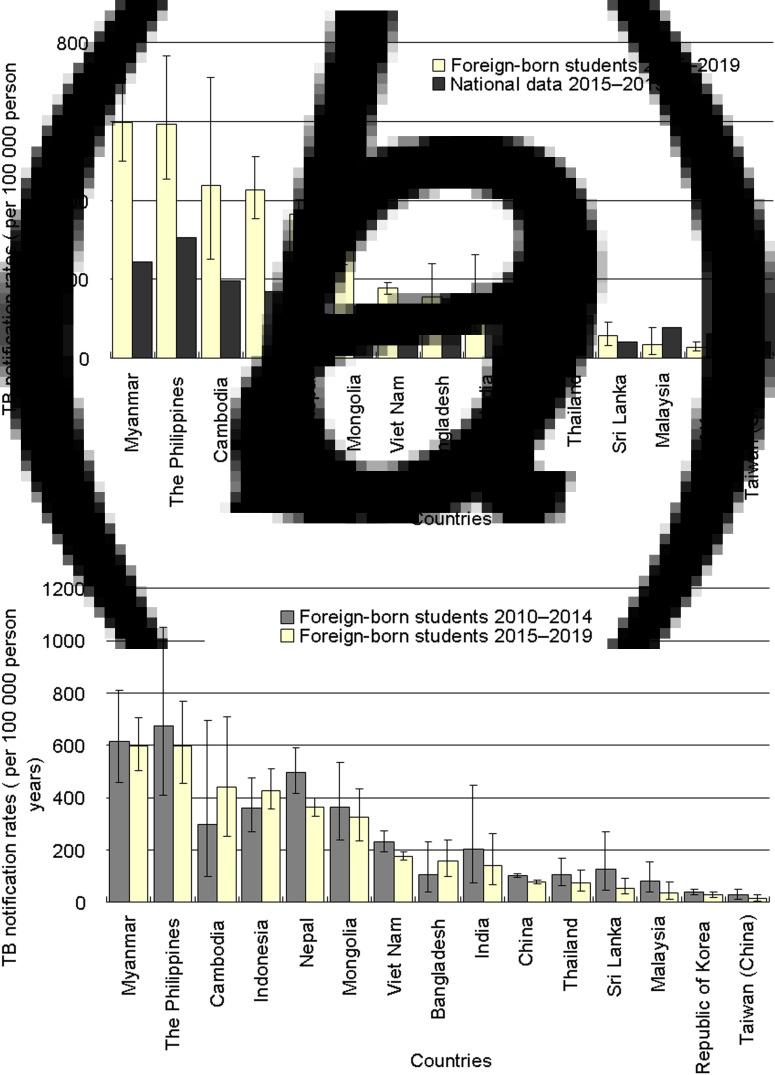
Tuberculosis notification rates of foreign students in Japan in 2015–2019 compared with those of their countries of origin (a) and those of foreign students in Japan in 2010–2014 (b). Error bars indicate 95% CIs. TB = tuberculosis. The data for 2010–2014 were derived from the authors' previous study [16].

The TB notification rates of the foreign students from Myanmar (596.6 per 100 000 PYs, 95% CI 501.1–704.9), the Philippines (595.4, 95% CI 453.6–767.4), Cambodia (438.6, 95% CI 250.9–711.3), Indonesia (427.4, 95% CI 355.1–510.1), Nepal (364.0, 95% CI 329.0–401.6) and Mongolia (324.8, 95% CI 236.1–435.8) were higher than 200 per 100 000 PYs (Fig. 3a). In addition, the TB notification rates of the foreign students were higher than those of the general populations for Myanmar (596.6 *vs.* 244.7 per 100 000 PYs), the Philippines (595.4 *vs.* 304.7), Cambodia (438.6 *vs.* 195.9), Indonesia (427.4 *vs.* 170.4), Nepal (364.0 *vs.* 113.3), Mongolia (324.8 *vs.* 133.1), Vietnam (176.9 *vs.* 106.2) and China (78.1 *vs.* 54.5) (Fig. 3a). Pearson's correlation analysis revealed that there was a statistically significant association between the TB notification rate for the foreign students in Japan and that in the general population among the 15 countries (*r* = 0.91, 95% CI 0.73–0.97). The TB notification rates of the foreign students with TB disease registered in 2015–2019 were significantly lower than those registered in 2010–2014 among those from Nepal (364.0 *vs.* 497.8 per 100 000 PYs), Vietnam (176.9 *vs.* 231.3) and China (78.1 *vs.* 100.9) (Fig. 3b).

## Discussion

This is a second report on the epidemiology of TB in foreign students in Japan. Almost all the TB cases among the foreign students were found because most (about 70% [22]) educational institutions in Japan conduct entry health screening with chest X-rays mandated by the School Health and Safety Act of 1958 [27]. In addition, most public institutions do annual health screening with chest X-rays for every student. Our results showed that the TB notification rate increased from 2015 to 2016, then decreased towards 2019 because the TB notification rates of the foreign students from China, Nepal and Vietnam, the three major countries of origin of the immigrants to Japan, decreased in that five-year period [24]. It was also reported that the approval rate for student visas dropped in late 2018 for students from Bangladesh, Myanmar, Nepal and Sri Lanka for fear of overstay reported in previous year [28]. This might have influenced the decrease in the TB notification rates. However, overall, the numbers of foreign students continuously increased from 2015 to 2019 (208 379, 239 287, 267 042, 298 980 and 312 214) [22].

The TB notification rates of the foreign students from several countries were higher than those of the general populations of their countries of origin probably because the NTPs of many countries might be unable to capture all the TB cases due to insufficient technical and managerial capacity, or as a result of TB patients' tendency to seek health care from the private sector and underreporting from that sector. A previous study found a high (more than 500 per 100 000 population) prevalence of smear-negative TB among immigrants in the United States of America who were born in China, the Philippines and Vietnam [29].

It also happened that some of foreign students shared a small apartment room with multiple roommates and were infected with TB from a roommate, and eventually developed TB disease after arriving in Japan [14]. According to other JASSO surveys on the life of the self-sponsored foreign students (*N* = 7025) in Japan in 2019 [30], 4086 (58.2%) students lived alone, whereas 1116 (15.9%) shared with another person, 1087 (15.5%) with two persons, and 736 (10.5%) with three or more persons. Regarding the space of the accommodation per person occupied by the students, most (4043, 57.6%) lived in the space over 10.0 m^2^ and 1344 (19.1%) lived in the space of 7.5–10.0 m^2^, whereas 1174 (16.7%) 5.0–7.5  m^2^ and 464 (6.6%) less than 5 m^2^. From the experience of one of the authors (MO) in his medical school years (late 1980s to early 1990s) in Japan, when he lived individually in a room with about 10.0 m^2^, a space less than 7.5 m^2^ per person is small for students.

The TB notification rates of the foreign students from China, Nepal and Vietnam decreased in 2015–2019 compared to 2010–2014 probably because, as we mentioned above, the incidences of TB of the general populations in the three countries decreased [24]. It might also be possible that foreign students from these countries voluntarily went through health screening before arriving in Japan because there have been many reports of TB outbreaks involving immigrant populations in Japan [14, 31, 32].

The median age of the student TB patients is low (22.5) because most foreign students coming to Japan are young. Since the JASSO surveys did not provide age distribution of foreign students in Japan, we have to estimate it from the proportions of foreign students attending various types of schools. The proportions of undergraduate and vocational school students are 29.5% and 22.2%, respectively [22], indicating over half of foreign students are probably aged between 18 and 22 years. The proportions of post graduate school students and Japanese language school students are 17.7% and 28.4% [22], respectively.

The findings of this study are similar to those of our previous study as the TB notification rates among the foreign students from countries such as Myanmar, the Philippines, Nepal and Mongolia were over 200 per 100 000 PYs [16], probably because the foreign students studying in Japan come from similar subpopulations of their countries of origin. The authors believe it is useful to continuously monitor the TB notification rates among the foreign students in Japan. The TB notification rates for the foreign students in Japan from most countries we investigated were higher than those of the general populations in their countries of origin in the previous study as well [16]. We previously investigated the positivity of the interferon-*γ* release assay (IGRA) among foreigners in Japan and the findings may support our current study: the IGRA positivity was well over 20% in those from Myanmar and Nepal, whereas it was not very high (about 3.0–6.0%) in those from China, the Republic of Korea, Sri Lanka, Taiwan and Vietnam [33], probably reflecting the variation of TB risks among these countries. Kuan reported the TB notification rates of labour- and marriage-related immigrants to Taiwan from China, Indonesia, the Philippines, Thailand and Vietnam and the rates were much lower, being about 10 (marriage-related immigrants from China) to 150 (labour-related immigrants from Indonesia) per 100 000 population [34]. This is probably because Taiwan requires visa applicants for internship and foreign spouses of health certificates [35], including chest X-ray findings [36], whereas Japan so far does not require any visa applicants of health certificates.

There are a few limitations in this study. First, some cases among the foreign students who were diagnosed with TB might not be incident TB cases but prevalent TB cases, i.e. they may have already developed TB disease before they arrived in Japan. However, considering that at least one-third of the foreign students with TB disease in our study themselves sought health care, they must be incident cases. It is not difficult for foreign students to seek care in Japan. All the government established universities have health centres in which students can seek care for free or with a small amount of cost (5–20 US dollars, depending on tests and care provided) per visit. In addition, long-term residents in Japan, including foreign students, are required to apply for health insurance with the minimum annual fee of about 165 US dollars, depending on their municipality of residence, their employment status and income. Also, they have to pay 30% of the total medical cost to the health care facilities from which they obtain the care. One can seek care from any health care facilities under the national health insurance scheme, even from tertiary or university hospitals with an additional cost (40–200 US dollars, depending on the institution) at the first visit, without being waitlisted for long time. It is also speculated that the proportion of prevalent TB is small given that cumulative lifetime risk of latent tuberculosis infection (LTBI) reactivation [37].

Second, our data derived from the national infectious disease surveillance system and the detailed information on the individual cases are quite limited. For example, some of the foreign students with TB disease might have been infected with TB in Japan, not in their countries of origin, and then might have developed TB disease, as mentioned above [14]. Although the evidence is scarce, molecular epidemiological studies showed that *Mycobacterium tuberculosis* isolates from foreign-born patients in Japan had more genetic diversity [38] and the proportion of TB caused by the recent transmission in foreign-born individuals in Japan was very small (1.8%) [39]. Another example may be that for about 4% of the foreign students the countries of origin were missing, leading to underestimation of the TB notification rates for some countries. We believe this is an inevitable limitation in relation to an investigation using the surveillance data.

Third, the denominator data, i.e. the numbers of foreign students by year, country and sex, were derived from JASSO and there might possibly be misclassification or missing data. Again, this is an inevitable limitation using statistical data collected by another organisation.

There are some implications from the findings of our study. First, the TB notification rates for the foreign students in Japan should continuously be monitored as it is a useful indicator of the risk of TB among the immigrant subpopulation in Japan. Second, immigrants should be provided with an information kit on TB and other communicable diseases, including health care resources available in Japan and an advice that they should seek health care if they have a cough for 2 weeks or more. Third, the immigrants from the countries with the high TB notification rates found in our study should annually be screened for TB, using chest X-ray, if deemed necessary, to detect TB disease, put them on anti-TB treatment early, and to ultimately prevent TB outbreaks in Japan. Fourth, the government of Japan is going to introduce pre-entry screening for TB of foreign individuals from selected countries who will stay in Japan for more than 6 months. The countries should be selected based on the risk of TB disease and our estimates should be considered in that selection. Fifth, in the pre-entry screening, IGRA tests may be conducted to screen LTBI and those who are IGRA-positive may possibly be offered for prophylaxis using isoniazid or rifampicin.

## Data Availability

The surveillance data for TB among foreign students in Japan are available as the supplement material.

## Supplementary material

For supplementary material accompanying this paper visit http://dx.doi.org/10.1017/S0950268821001977. click here to view supplementary material
